# Water as a Probe for Standardization of Near-Infrared Spectra by Mutual–Individual Factor Analysis

**DOI:** 10.3390/molecules27186069

**Published:** 2022-09-17

**Authors:** Xiaoyu Cui

**Affiliations:** BIC-ESAT and SKL-ESPC, College of Environmental Sciences and Engineering, Peking University, Beijing 100871, China; xycui@pku.edu.cn

**Keywords:** water probe, mutual–individual factor analysis, calibration transfer, aquaphotomics, near infrared spectroscopy

## Abstract

The standardization of near-infrared (NIR) spectra is essential in practical applications, because various instruments are generally employed. However, standardization is challenging due to numerous perturbations, such as the instruments, testing environments, and sample compositions. In order to explain the spectral changes caused by the various perturbations, a two-step standardization technique was presented in this work called mutual–individual factor analysis (MIFA). Taking advantage of the sensitivity of a water probe to perturbations, the spectral information from a water spectral region was gradually divided into mutual and individual parts. With aquaphotomics expertise, it can be found that the mutual part described the overall spectral features among instruments, whereas the individual part depicted the difference of component structural changes in the sample caused by operation and the measurement conditions. Furthermore, the spectral difference was adjusted by the coefficients in both parts. The effectiveness of the method was assessed by using two NIR datasets of corn and wheat, respectively. The results showed that the standardized spectra can be successfully predicted by using the partial least squares (PLS) models developed with the spectra from the reference instrument. Consequently, the MIFA offers a viable solution to standardize the spectra obtained from several instruments when measurements are affected by multiple factors.

## 1. Introduction

Water, as one of the most common substances on earth, has numerous functions, including dissolution, stabilization, catalyzation, transportation, etc. [1,2,3]. However, water is an enduring mystery, due to the intricate and dynamic structures of its intermolecular hydrogen bond network [4]. The molecular interaction is generally affected by perturbations in water’s surroundings, such as temperature and additives, resulting in hydrogen bond rearrangement and the alteration of chemical as well as physical performance in the system [5]. Thus, water structure has remained a significant research subject for decades.

Aquaphotomics has been proposed as a new scientific discipline based on innovative knowledge of the water molecular network, which describes the features of water structure from the water spectrum, indirectly reflecting all perturbations, including experimental conditions and sample compositions [6]. The water spectral pattern hence becomes a revelator of the system condition. Several attempts have been made to analyze the experimental conditions by using water spectra. Shao et al. reported that a quantitative spectra–temperature relationship (QSTR) model can be established, and the temperature of a solution can be predicted from the near-infrared (NIR) spectrum with the water region using the model [7,8]. Romanenko et al. developed a new approach, which used three fitted Gaussian features from the water Raman spectra to investigate pressure and solution density [9]. Subsequently, aquaphotomics has gradually broadened as water was applied to be a sensor and an amplifier in the structural and quantitative analysis of aqueous systems [10]. A number of researchers have reported that water was a sensitive probe for analyzing the structural changes and the interactions in aqueous solutions of alcohols [11,12], as well as proteins [13,14,15], and practical samples [16,17,18]. On the other hand, the water probe possesses its own advantages when carrying out the component detection [19,20,21].

Currently, aquaphotomics for experimental conditions and sample compositions in aqueous systems have been investigated simultaneously with chemometric methods [22,23,24]. The NIR spectra of water in terms of hydrogen bonding were extracted to characterize the perturbations created by the change of temperature and concentration in solutions with the application of multivariate curve resolution–alternating least squares (MCR–ALS) [25], alternating trilinear decomposition (ATLD) [26], and multilevel simultaneous component analysis (MSCA) [27], etc. Furthermore, practical samples were studied [28,29]. The common spectral features that contained the water spectra influenced by temperature were extracted from the spectra of serum samples, and both the temperature and glucose can be successfully measured by using mutual factor analysis (MFA) [29]. Therefore, aquaphotomics provides a common platform for practical applications.

In industrial applications with NIR spectroscopy, standardization or calibration transfer is usually required to correct the spectral variations both caused by the measurement and sample conditions [30]. There are three major pathways for standardizing the NIR spectra measured on different instruments, including the correction of the prediction values [31], alteration of the model coefficients [32,33], and modification of the spectra [34,35,36,37]. The last strategy is the most commonly applied. Piecewise direct standardization (PDS) is a very efficient method by which to establish a linear relationship between the spectra measured on different instruments in several small window regions [34]. By using techniques like spectral space transformation (SST) [35], alternating trilinear decomposition (ATLD) [36], and multilevel simultaneous component analysis (MSCA) [37], research was also conducted by determining the relationship between the principal components retrieved from the spectra as an alternative to correcting spectra. However, the majority of standardization methods concentrate on resolving the discrepancy resulting from a straightforward link between the spectra obtained on various instruments and describing the calibration transfer through a mathematical formula. Aquaphotomics may provide a different approach by which to examine spectral differences that are influenced by a variety of circumstances, enhancing interpretability and lowering bias, especially for the spectra of samples containing water, such as agricultural products.

In this work, particular attention is paid to the application of aquaphotomics for calibration transfer to discover more information with physical and chemical meanings. A new algorithm, called mutual–individual factor analysis (MIFA), using water as a probe, was proposed to analyze the water spectra both influenced by different instruments and substances, and the standardization performance of the water probe was validated by two NIR spectral datasets.

## 2. Theory and Algorithm

### 2.1. Continuous Wavelet Transform

As an efficient tool for data processing, continuous wavelet transform (CWT) has been generally applied to improve the spectral quality, i.e., resolution enhancement, baseline correction, and smoothing [29,38,39]. In this work, CWT with a Symmlet filter with a vanishing moment 6 (Sym6 filter)) was employed, which is approximately equal to the sixth derivative. With CWT, the resolution improvement and spectrum smoothing can be achieved simultaneously [39]. Due to the property of the sixth derivative, the positive absorption in the raw spectra becomes a negative one. In this study, for the convenience of description, the value of the derivative is reversed.

### 2.2. Mutual–Individual Factor Analysis

In order to develop the transfer model, the spectra of standard samples collected on different instruments are generally employed for standardization. Following CWT processing, the spectra from three instruments, denoted as **X**_1_, **X**_2_, and **X**_3_ were employed in this investigation. In accordance with MFA [29], the combined spectral matrix, **X***_comb_*, was first processed to separate the standardized signal (**SS)**, which represents the spectral information of samples measured on a reference instrument. The relationship can be presented as

(1) $$X_{comb}=\left[X_{1},X_{2},X_{3}\right]=T\left[P_{1}^{T},P_{2}^{T},P_{3}^{T}\right]+E$$

(2) $$SS=TP_{ref}^{T}=X_{ref}{\left(P_{ref}^{T}\right)}^{+}P_{ref}^{T},$$

where superscripts T and + denote the mathematical operation of transposition and pseudoinverse of the matrix, respectively, and **E** contains the residuals between the actual spectra and the fitted model. The scores and loadings in a principal component analysis (PCA) model are symbolized as **T** and **P***_i_*^T^ (*i* = 1, 2, and 3), respectively. As a result of using the same **T**, **P***_i_*^T^ represents the variations in the measured conditions or instruments. Then, it is possible to determine how much of the spectral pattern of **X***_i_* is present in **X***_j_* (*i* ≠ *j*) by using the relationship. The relative quantity (*z_i_*) of **SS** contained in each **X***_i_* can be obtained, and score, **t***_i_*, in each group of spectra collected on different instruments can be calculated by using the reference loading, **P***_ref_*. The relationship can be presented as

(3) $$z_{i}=trace\left(X_{i}SS^{+}\right)$$

(4) $$t_{i}=X_{i}{\left(P_{ref}^{T}\right)}^{+}.$$

It should be noted that, theoretically, any group of spectra measured on an instrument can be used as the reference; however, generally the spectra collected on the master instrument is applied.

The mutual part between the spectra measured on various instruments can be discovered after MFA processing. However, apart from the spectral alterations driven by the instruments, there are also spectral variations caused by the altered sample structural features as a result of changing measurement conditions. Despite being minor, the spectral variations affect the standardization, especially for the samples containing water, due to the sensitive response of OH in NIR spectra [6]. As a result, the individual factor was introduced to evaluate the variance using PCA model as the following equation,

(5) $$X_{lef}=\left[\begin{array}{c} X_{1}-z_{1}SS^{+}\\ X_{2}-z_{2}SS^{+}\\ X_{3}-z_{3}SS^{+} \end{array}\right]=\left[\begin{array}{c} T_{lef,1}\\ T_{lef,2}\\ T_{lef,3} \end{array}\right]P_{lef}^{T}+E_{lef},$$

where **E***_lef_* denotes the remaining data that did not fit into the model, and **T***_lef,i_* and **P***_lef_*^T^ reflect the scores and loadings in the PCA model, respectively, after the mutual parts have been removed. As a result, the score of the model merely accounts for the variance affected by the measurement of the sample. Because the essence of the algorithm is to extract the factor mutually and individually contained in the spectral data of different samples, the algorithm is named as mutual–individual factor analysis and abbreviated as MIFA.

By adjusting the coefficients, i.e., *z_i_* and **T***_lef,i_*, the spectra measured on one instrument can be transferred to another. The details of the standardization can be summarized in two steps, as follows.

(1) Establish MIFA models: The MIFA approach is applied to establish the two models, containing **t***_i_***, P***_i_*^T^, **T***_lef,i_*, and **P***_lef_*^T^, from the standard spectra measured on three instruments, **X**_1_, **X**_2_, and **X**_3_. This stage involves determining how many principal components each of the two models has.

(2) Transfer the spectrum: As an illustration, the following computations can be used to transfer the spectrum of instrument 2 (**X**_2*s*_) to instrument 1,

(6) $$t_{s}=X_{2s}{\left(P_{1}^{T}\right)}^{+}$$

(7) $$T_{lef,s}=(X_{2s}-t_{s}P_{1}^{T}){\left(P_{lef}^{T}\right)}^{+}$$

(8) $$t_{Ts}=t_{s}{\left(t_{2}\right)}^{+}t_{1}$$

(9) $$T_{lef,Ts}=T_{lef,s}{\left(T_{lef,1}\right)}^{+}T_{lef,2}$$

(10) $$X_{T2s}=t_{Ts}P_{1}^{T}+T_{lef,Ts}P_{lef}^{T}.$$

The scores of **X**_2*s*_ can be calculated by using Equations (6) and (7) through MIFA models, and then transferred from instrument 2 to instrument 1 by using Equations (8) and (9). Finally, Equation (10) can be applied to obtain the transferred spectra by using the loadings (**P**_1_^T^ and **P***_lef_*^T^) and the transferred scores. The spectra from the instrument 3 can be transferred in the same way.

## 3. Data Description

Two NIR spectral datasets were employed in this investigation. Dataset 1 was downloaded from http://software.eigenvector.com/Data/Corn/index.html (accessed on 25 June 2019), and contains the moisture, oil, protein, and starch contents of 80 corn samples along with the NIR spectra obtained by using three NIR spectrometers (m5, mp5, and mp6). Each spectrum was acquired with 700 data points throughout the wavelength range of 1100–2498 nm with a digitization interval of 2 nm.

Dataset 2 was downloaded from https://www.cnirs.org/content.aspx?page_id=22& club_id=409746&module_id=239453 (accessed on 17 July 2022), and includes 744 NIR spectra analyzed on three instruments (A1, A2, and A3), as well as the protein content of the 248 wheat samples. Each spectrum comprised 741 data points with a digitization interval of 0.5 nm, and was recorded in the wavelength range of 730–1100 nm.

Prior to the calculation, each dataset was divided into a calibration set, a transfer set, and a prediction set using the Kennard-Stone (KS) technique [40]. The calibration set (the spectra of the master) is employed to develop the multivariate calibration model of the master, the transfer set (containing the spectra of all the instruments) is applied to building the transfer model, and the prediction set (the spectra of the salve) is used for validating the effect of the transfer model. For dataset 1, the calibration, transfer, and prediction sets were composed of 30, 30, and 20. For dataset 2, the possible outlier (ID 20140190) was removed, and the remaining 247 samples were divided into a calibration set of 117 samples, a transfer set of 30 samples, and a prediction set of 100 samples.

## 4. Results and Discussion

### 4.1. Spectral Analysis and Resolution Enhancement

**Figure 1:**
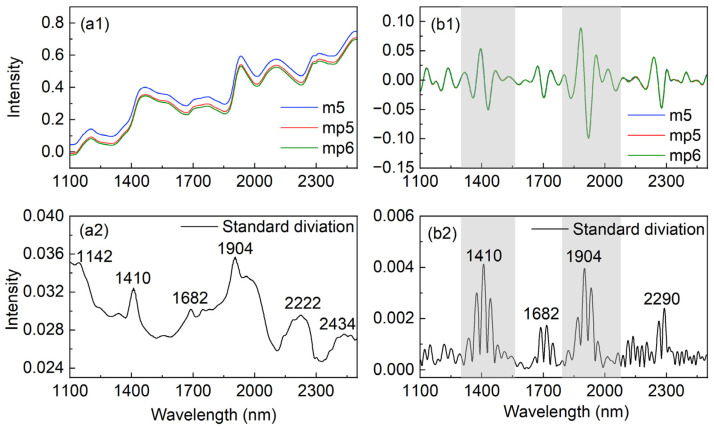
Average NIR spectra of corn samples from instrument m5, mp5, and mp6, respectively (**a1**), standard deviation of the averaged spectra (**a2**), average spectra from each instrument after CWT transformation (**b1**), and standard deviation of the CWT processed spectra (**b2**). The selected ranges were indicated with shadows.

The spectra of corn samples using three instruments are displayed in Figure 1. Figure 1(a1) shows that there are several broad bands with a ranked background, and the average spectrum intensity from m5 is higher than those from mp5 and mp6. The outcome demonstrates that the background shift appears to be the major source of the overall spectral variance induced by different instruments. To remove the background, CWT with Sym6 was used, which is approximately equal to the sixth derivative [39]. The CWT-processed spectra are shown in Figure 1(b1), illustrating an almost zero baseline. Furthermore, compared with the spectra from the three instruments, the similarity proves that the background shifting among the instruments is the primary cause of the difference. In addition, taking advantage of the high-order derivative, narrower peaks were obtained than the corresponding spectra in Figure 1(a1). It has been reported that compared with the results by the first or second derivatives used in our previous works [11,17,26,28], the CWT-processed spectra from higher-order derivatives illustrate higher resolution, and reveal more information to understand the interactions in aqueous samples [29,41].

For a further comparison, Figure 1(a2,b2) provides the standard deviation of the sample spectra and the CWT-processed spectra, respectively, where the higher the intensity, the larger the difference. The relatively higher intensity in both subfigures can be seen around 1410 and 1904 nm, which are primarily composed of the overtone and a combination of stretching and bending vibrational modes of OH in water, as well as features of OH, NH, and CH in biological components [6,18]. The others around 1682 and 2200–2300 nm are related to the CH groups in biomolecules and α-helix in protein, respectively, consistent with the assignments of NIR bands from quantum chemical simulations [42,43]. Additionally, the background shift may be the reason of the high intensity around 1142 and 2434 nm in Figure 1(a2), as there are no strong values in these spectral regions of Figure 1(b2). These findings suggest that as detecting conditions change, different instruments alter not just the background but also the spectral intensity of various species in samples, which is consistent with the results of MSCA [37]. Thus, it is necessary to adjust the spectra at both the instrument and sample levels.

Furthermore, the major differences in the shadow reveal that NIR spectra are sensitive to OH, which can easily undergo structural changes due to hydrogen bonding when environmental changes are detected [6,24]. Compared with biomacromolecules, such as oil, protein, and starch in seeds, OH groups in water are relatively more active due to the smaller size and stronger polarity of water, and may cause more spectral changes from different instruments [6,44]. For this reason, by using water as the probe, the spectral ranges (1294–1556 and 1788–2078 nm) associated with water in the shadow in Figure 1(b1) were chosen to build the MIFA model.

### 4.2. Mutual–Individual Factor Analysis

To investigate the instrument and sample effects on the NIR spectra, MIFA was employed on dataset 1 using water as a probe. First, the numbers of principal components (PC) were required to construct the mutual and individual factor analysis models, respectively. The commonly used criteria of “explained variance” was utilized in this study to determine the numbers [37]. The number of the PCs that explain 99.9% of the variance was applied. For dataset 1, the parameters for the mutual and individual factor analysis models were 1 and 6, respectively.

**Figure 2:**
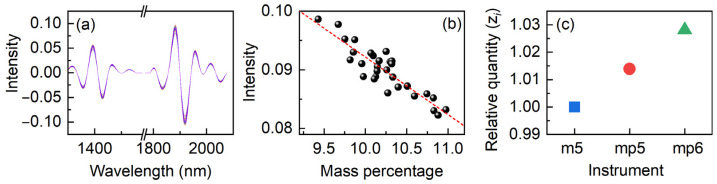
Standardized signal (**SS**) (**a**), the relationship between the intensity at 1882 nm and the mass percentage of moisture (**b**), and the relationship between the relative quantity (*z_i_*) and instruments (**c**). The linear regression appears as the red dash.

Equations (1)–(3) were then applied to calculate the standardized signal (**SS**) and the relative quantity (*z_i_*) using the spectra from m5 as the reference. The **SS** is shown in Figure 2a to estimate instrument-induced variance. As a result, it should be unaffected by instrument changes. The intensity of the signal should be only related to the concentration of the samples. Figure 2b shows the intensity variations at 1882 nm with the mass percentage of moisture to validate the assumption. A linear function can be generated from quantitative analysis of complicated biological samples with a recovery of less than 20%, which is considered a good result when using single-point spectral values for regression. The results suggest that **SS** is related to water in corn, indicating the effectiveness of mutual parts, which are consistent with the MFA conclusion [29].

Figure 2c depicts *z_i_* in relation to the three instruments. The very small difference in values reflects the minor overall variations between the spectra of three instruments, even if the background was reduced by the CWT. This demonstrates that apart from the background variation, the mutual part contains the sample change with different instruments, indicating the sensitivity of water probe. *z_i_* is a mirror of the spectral variation caused by instruments; hence the relationship between *z_i_* can be utilized to regulate the overall spectral difference caused by the measurement circumstances.

**Figure 3:**
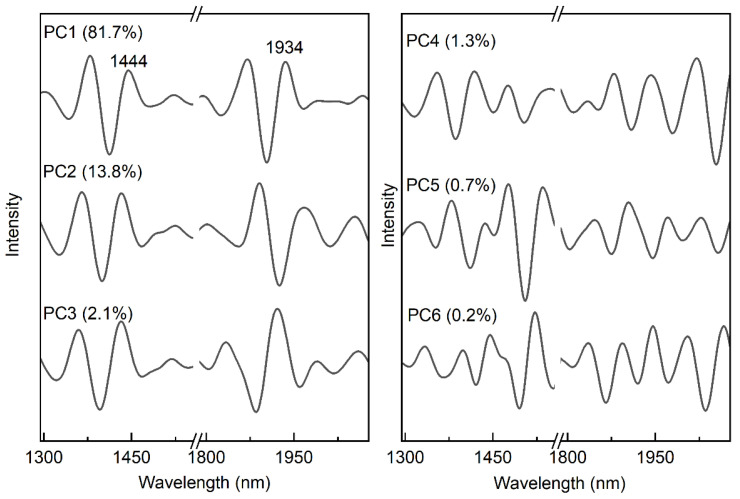
The first through the sixth loadings in the PCA model, after the mutual parts have been removed.

After removing the mutual part from the spectra, PCA was used to evaluate the individual spectral features from the remaining spectra. Figure 3 shows the loadings for each PC, and the spectrum characteristics are related to the hydrated CH, NH, and OH in biomolecules as well as the hydrated OH in water [6,15,16,18,42,43]. The disparities between the PCs may be due to differences in the samples and the measurement of the spectra. In comparison to the other five PCs, the first PC provides additional details concerning the characteristics of the hydrogen-bonded OH at 1444 and 1934 nm, according to aquaphotomics [10]. When samples of corn are measured by using different instruments, the outcomes show that the hydrogen-bonding variance of the chemicals in the corn is what causes the majority of individual differences.

**Figure 4:**
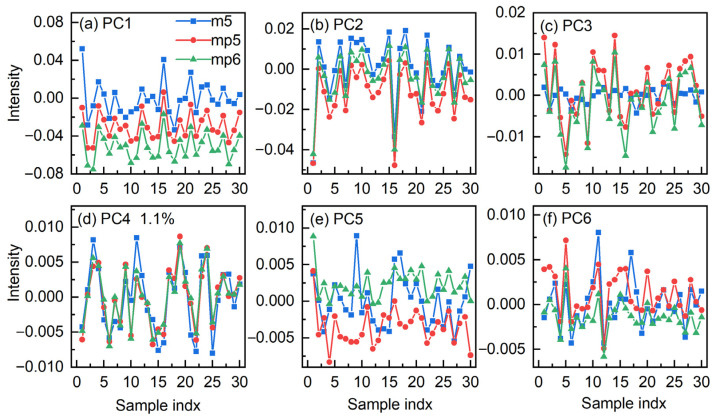
Comparison of the scores in the first through the sixth (**a**–**f**) PCs in the individual part for the spectra from the instruments m5, mp5, and mp6. The blue square, red circle, and green triangle represent the score for instrument m5, mp5, and mp6, respectively.

In order to further explore the feasibility of the model transfer, Figure 4 displays the scores of the individual part for the first six PCs. The scores in the models are more essential in this study because the transfer is accomplished by modifying the scores. This implies that measurement-related changes in molecule structures may impact the measured spectra, as not all of the discrepancy can be accounted for by the mutual part or only one model [37]. For adjusting the complicated effects, a multi-step strategy can be a wise solution.

In contrast to the scores in PC2–PC6, it is obvious that PC1 shows the largest disparity. In PC1, the scores from mp5 and mp6 show similar changes when compared with that from m5, indicating that structural alterations of hydrogen-bonded water in corn have a regular pattern under different measurement conditions. The findings prove that water probe may be utilized for model transfer and that water spectral features can be used as a comprehensive descriptor to portray the system.

### 4.3. Standardization of the Spectra

By adjusting the coefficient values in the scores, it is possible to achieve spectral standardization, or the transfer of the spectra from mp5 and mp6 to m5 (mp5–m5 and mp6–m5). Because there is just one value for the mutual part in dataset 1, it is obvious that the transfer may be finished by simply changing the *z_i_* of mp5 and mp6 to m5, respectively. In general, the transfer for the mutual part can be accomplished by Equation (8). Similarly, Equation (9) can be used to determine the transfer of individual part.

**Figure 5:**
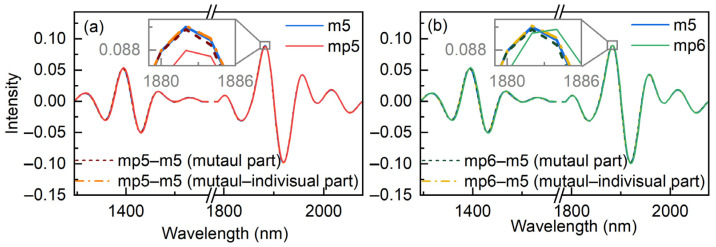
The transfer results for an arbitrarily selected spectrum from mp5 (**a**) and mp6 (**b**) to m5, respectively.

Figure 5 displays the spectra measured from m5, mp5, and mp6 for a sample randomly chosen from the prediction set of dataset 1. The transferred spectra by the mutual and mutual–individual parts are also shown in the figure to demonstrate the effects of the standardization by the proposed strategy. Clearly, the spectra from the three instruments differ among one another in the embedded graphs. The spectra of mp5 and mp6 approach closer to the spectrum of m5 once the mutual part has been corrected, although there is still a little divergence. The spectra from m5, mp5–m5, and mp6–m5 are virtually identical after the individual parts have been corrected. The findings unequivocally demonstrate that both mutual and individual parts have an impact on the transfer of the spectra.

**Figure 6:**
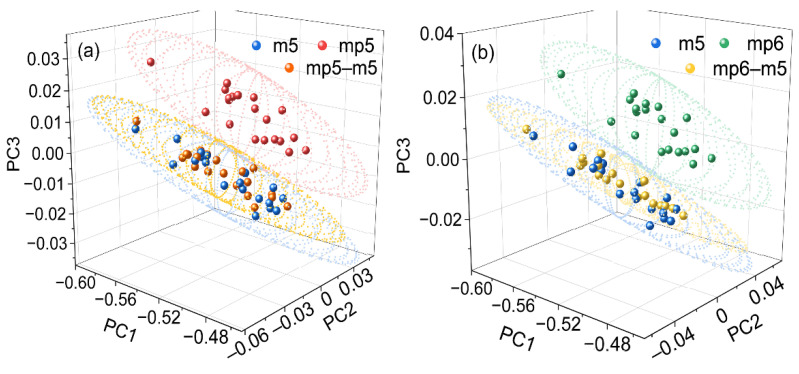
Results of the scores in PC1–PC2–PC3 space for the spectra from m5, mp5, and mp5–m5 (**a**) and spectra from m5, mp6, and mp6–m5 (**b**), respectively. The ellipsoids are determined with the confidence of 95%.

To further assess the transfer effect of the proposed approach, PCA was carried out on the spectra both before and after spectral standardization. Figure 6 displays the transfer outcomes of the spectra from various equipment in the first three PC spaces. The scores from m5 and the other two instruments differ substantially, demonstrating the variation in spectra between them. The disparity between the mp5 and mp6 is not as significant, consistent with the findings in Figure 1. The results show that the score can properly represent the spectral features.

When the scores of the reference and the transferred spectra are compared, it is clear that the ellipsoids with a confidence value of 95% are overlapped, demonstrating that the disparity has been corrected by MIFA. The outcomes unequivocally illustrate that the proposed method is capable of transferring the measured spectra from other instruments to the reference.

### 4.4. Validation of the Standardized Spectra

**Figure 7:**
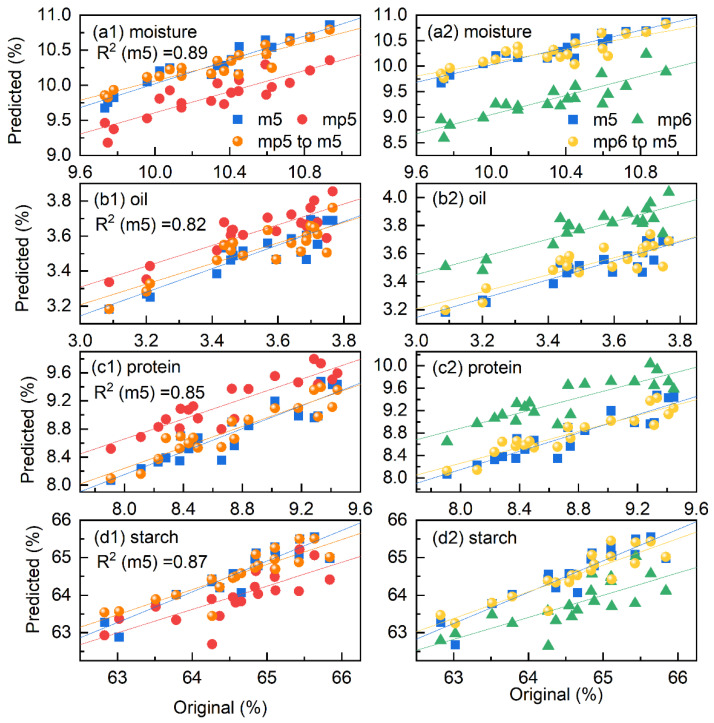
Relationship between the original and the prediction values of moisture (**a**), oil (**b**), protein (**c**), and starch (**d**) obtained from the spectra from m5, mp5, and mp6, as well as the transferred spectra of mp5–m5 and mp6–m5, respectively.

For the final evaluation of the proposed method, a partial least squares (PLS) model was built with the calibration spectra measured on the reference instrument, and then applied to the prediction set from other instruments. Cross-validation is used to establish the optimal number of latent variables (*n*LV) in the PLS model. For the calibration set of dataset 1, four LVs were employed. Figure 7 depicts the relationship between predicted and original moisture, oil, protein, and starch values based on the spectra from m5 in blue points. The results of the spectra from mp5 and mp6 are displayed in red and green points, and the predicted values of the transferred spectra from mp5 and mp6 are also plotted in orange and yellow for comparison. It is obvious that similar results can be found between the blue and orange or blue and yellow points in the subfigures, respectively. Moreover, the blue and red or blue and green points clearly differ from each other. The results demonstrate that the PLS model from the reference instrument can accurately predict the transferred spectra.

When comparing the correlation coefficient (R^2^) of the calibration models, it should be noted that the relationships between the original and predicted values for moisture, protein, and starch are slightly better than that for oil. The moisture quantification result is the best because the water spectral range was chosen. In addition, protein and starch, which interact more strongly with water [2,10], have superior quantification models than oil. Despite the fact that R^2^ is slightly lower than others for oil, the quantitative model can be applied for practical samples with the recovery less than 20%.

To further investigate the effectiveness of the proposed approach, Table 1 and Table 2 exhibit the predictions made by MIFA for datasets 1 and 2, respectively, compared with the results from PDS and SST. By using one of the instruments as a reference, the values of root mean squared error of prediction (RMSEP) for the oil and protein contents in the validation set are shown in the tables, respectively. Clearly, the direct prediction of spectra from other instruments through the reference model is substantially worse than that of the reference spectra. The RMSEP can be minimized if the transferred spectra are predicted. The results provide a strong validation for the efficiency of the calibration transfer, even though the values are still slightly larger than those of the reference instrument. Additionally, although there is a minor variance, similar results are also obtained for the PDS, SST, and MIFA. Consequently, the MIFA can be used to successfully transfer the spectra measured by different instruments with the water spectral region. Furthermore, the MIFA approach (using water as a probe) narrows the wavelength range needed by concentrating on the water spectral region, which reflects the major structural changes in the system, providing the opportunity of standardization between miniature instruments.

## 5. Conclusions

For the purpose of standardizing NIR spectra, a new chemometric technique called mutual–individual factor analysis (MIFA) was developed based on the water spectral region, which used water as a probe. In order to describe the overall differences between the various instruments, the method extracted the spectral feature of the mutual part present in the spectra from different instruments. The difference between the molecular interactions in the samples caused by various measurement conditions was then depicted in each individual part. Furthermore, the spectra measured on one instrument can be effectively transferred to that of another by modifying the coefficients of the mutual and individual parts, respectively. When compared with PDS and SST, MIFA produced a similar result, but provided additional information with physical and chemical meanings by using aquaphotomics. Therefore, in practical applications of NIR spectroscopic analysis, the water probe may offer an effective solution when the spectra are impacted by several complex perturbations, and promote the development of small instruments with limited wavelength ranges.

## Figures and Tables

**Table 1:** Comparison of the results obtained by PDS, SST and MIFA for dataset 1.

Calibration Spectra	Validation Spectra	RMSEP
m5	m5	0.1419
	mp5	0.1667
	mp6	0.2608
	mp5–m5 (MIFA)	0.1435
	mp5–m5 (PDS)	0.1592
	mp5–m5 (SST)	0.1573
	mp6–m5 (MIFA)	0.1424
	mp6–m5 (PDS)	0.1519
	mp6–m5 (SST)	0.1493
mp5	mp5	0.1527
	m5	0.1786
	mp6	0.1669
	m5–mp5 (MIFA)	0.1601
	m5–mp5 (PDS)	0.1642
	m5–mp5 (SST)	0.1639
	mp6–mp5 (MIFA)	0.1546
	mp6–mp5 (PDS)	0.1593
	mp6–mp5 (SST)	0.1572
mp6	mp6	0.1523
	m5	0.1981
	mp5	0.1909
	m5–mp6 (MIFA)	0.1609
	m5–mp6 (PDS)	0.1564
	m5–mp6 (SST)	0.1551
	mp5–mp6 (MIFA)	0.1546
	mp5–mp6 (PDS)	0.1634
	mp5–mp6 (SST)	0.1586

**Table 2:** Comparison of the results obtained by PDS, SST and MIFA for dataset 2.

Calibration Spectra ^1,2^	Validation Spectra	RMSEP
A1	A1	0.6091
	A2	0.8028
	A3	0.9866
	A2–A1 (MIFA)	0.6824
	A2–A1 (PDS)	0.6987
	A2–A1 (SST)	0.6752
	A3–A1 (MIFA)	0.7154
	A3–A1 (PDS)	0.7089
	A3–A1 (SST)	0.7066
A2	A2	0.7475
	A1	0.8538
	A3	0.9274
	A1–A2 (MIFA)	0.8049
	A1–A2 (PDS)	0.8122
	A1–A2 (SST)	0.799
	A3–A2 (MIFA)	0.8106
	A3–A2 (PDS)	0.8324
	A3–A2 (SST)	0.8075
A3	A3	0.7044
	A1	0.8316
	A2	1.1068
	A1–A3 (MIFA)	0.7993
	A1–A3 (PDS)	0.8237
	A1–A3 (SST)	0.8033
	A2–A3 (MIFA)	0.8196
	A2–A3 (PDS)	0.8169
	A2–A3 (SST)	0.8127

^1^ The number of PC for the mutual and individual factor analysis models were 1 and 6 in MIFA, respectively. ^2^ PLS model was built with 3 LVs, according to cross-validation.

## Sample Availability

Samples of the compounds are not available from the authors.
